# Alterations in the glutathione metabolism could be implicated in the ischemia-induced small intestinal cell damage in horses

**DOI:** 10.1186/1746-6148-5-10

**Published:** 2009-03-18

**Authors:** Gonzalo Marañón, William Manley, Patricia Cayado, Cruz García, Mercedes Sánchez de la Muela, Elena Vara

**Affiliations:** 1 Horsepital SL, Villanueva del Pardillo, Madrid, Spain; 2 Department of Biochemistry and Molecular Biology, Medical School, University Complutense of Madrid, Ciudad Universitaria s/n, 28040 Madrid, Spain; 3 Department of Animal Medicine and Surgery, Veterinary School, University Complutense of Madrid, Ciudad Universitaria s/n, 28040 Madrid, Spain

## Abstract

**Background:**

Colic could be accompanied by changes in the morphology and physiology of organs and tissues, such as the intestine. This process might be, at least in part, due to the accumulation of oxidative damage induced by reactive oxygen (ROS) and reactive nitrogen species (RNS), secondary to intestinal ischemia. Glutathione (GSH), being the major intracellular thiol, provides protection against oxidative injury. The aim of this study was to investigate whether ischemia-induced intestinal injury could be related with alterations in GSH metabolism.

**Results:**

Ischemia induced a significant increase in lipid hydroperoxides, nitric oxide and carbon monoxide, and a reduction in reduced glutathione, and adenosine triphosphate (ATP) content, as well as in methionine-adenosyl-transferase and methyl-transferase activities.

**Conclusion:**

Our results suggest that ischemia induces harmful effects on equine small intestine, probably due to an increase in oxidative damage and proinflammatory molecules. This effect could be mediated, at least in part, by impairment in glutathione metabolism.

## Background

Colic refers to any cause of abdominal pain and is the leading cause of death in horses. Much of the mortality is associated with ischemic-injured intestine because of the rapid deterioration of the intestinal barrier, absorption of bacterial lipopolysacharide and subsequent circulatory collapse [1]. Certain processes that can take place during equine colic, like intestinal ischemia and/or endotoxemia [1-4] can subject the intestine to an increased burden of oxidative stress [4,5], with the subsequent accumulation of reactive oxygen species (ROS) and reactive nitrogen species (RNS). ROS are highly reactive molecules which are mainly generated in mitochondria during oxygen metabolism. Impairment in mitochondrial function may reduce the energy supply to the cells. It has been suggested that the increase in oxidative stress could be, at least in part, responsible for this fact [6].

Two molecules that have been recently involved in oxidative damage and inflammatory response are Nitric oxide (NO) and Carbon monoxide (CO). Nitric oxide can act as both inflammatory mediator and RNS, either directly or through peroxynitrites generated by its interaction with O_2_^- ^[7,8]. CO is one of the elements of the heme-oxygenase 1 (HO-1)-CO pathway, which has been proposed to constitute a defence system against oxidative and inflammatory damage [9].

In the past, many studies have been shown that glutathione (GSH), a well-known antioxidant [10,11] protects cells and organs from various forms of injury induced by hypoxia, ischemia, cold preservation and drugs [12,13]. This has been used as evidence for a role of oxygen free radicals in such injury because the metabolism of GSH suppresses the cytotoxic effects of the reactive radicals [14,15]. Nevertheless, GSH is also essential in preserving the ability of the cell to generate ATP and to maintain membrane integrity [13], and it has been suggested that GSH may protect cells by mechanisms independent of its antioxidant properties.

Oxidative stress secondary to free radical generation may play a role in the tissue damage associated with intestinal ischemia [5]. Mitochondria are major producers of ROS, i.e. O_2_^- ^and H_2_O_2_. H_2_O_2_if not reduced to water, can lead to the formation of very reactive hydroxyl radicals resulting in the formation of lipid hydroperoxides (LPO) that can damage mitochondria membranes and functions reducing the energy supply to the cells. Since mitochondria do not contain catalase, GSH as a cofactor of the glutathione peroxidase is the only mitochondrial defence to coupe with H_2_O_2 _produced endogenously in aerobic cells. Thus, GSH is critical in protecting unsaturated fatty acids in membrane phospholipids from peroxidation by attack of oxygen free radicals. GSH depletion results in cell injury and death in several cell types and organs [15,16] and it has been shown that glutathione is required for intestinal function [16], but, to our knowledge, no studies concerning the role of glutathione metabolism in horse intestine have been done.

On the other hand, S-Adenosyl methionine (SAMe) an endogenous metabolite synthesized in the cytosol of all types of cells from ATP and methionine [17], in a reaction catalyzed by the enzyme methionine-adenosyl-transferase (MAT), plays a critical role in the synthesis of glutathione [18,19]. SAMe plays a critical role in the synthesis of glutathione (GSH) [18,19]. Conversely, depletion of GSH could result in a decrease of MAT activity, which in turn can result in a further decrease of GSH levels thus increasing cell damage. SAMe also participates in polyamine synthesis and it acts as methyl donor for most biological transmethylation reactions, catalysed by methyl-transferases (MetTase), including the methylation of phosphatidyl ethanolamine to produce phosphatidyl choline (PC), which is an essential molecule for cell membrane integrity.

The aim of this study was to asses a possible association between alterations in the glutathione metabolism and intestinal ischemia-induced oxidative/antioxidative imbalance in horses. For this purpose GSH, LPO and ATP content, as well as NO and CO release was determined in intestinal tissue of horses. Additionally, MAT and Met Tase activities have also been investigated.

## Methods

### Patients

Twenty-nine adult horses with acute abdominal pain subjected to emergency abdominal surgery of the small intestine, referred to our clinic during the last year, were used. The ages ranged from 5 to 20 years with a mean of 11 years. On arrival, the same protocol for acute abdominal emergency reception was applied to each horse. A complete anamnesis was performed at time of presentation (duration of signs, previous medical treatment....), and a clinical evaluation to asses degree of pain, response to analgesia, abdominal distention, hydration status, rectal examination findings and amount of gastric reflux. Degree of abdominal pain was assessed by the attending clinicians as mild (occasional pawing, occasionally turning the head to the flank, stretching out), moderate (cramping with attempts to lie down, kicking at the abdomen, laying down and attempting to roll or rolling) or severe (sweating, dropping to the ground, violent rolling) [20].

At presentation, rectal temperature varied from 37.6 to 38.5°C (38.1 ± 0.7°C), heart rate ranged from 45 to 90 beats/min (68 ± 23 beats/min), and respiratory rate ranged from 10 to 30 breaths/min (20 ± 10 breaths/min). All of the 29 colic horses had moderate or severe pain. The response to analgesia was mild in 14 horses and poor in 15. Transrectal palpation of the abdominal organs was performed in all cases.

The cases were composed of one inguinal hernia, 5 lipomas, 2 intussusceptions (parasites), 1 horse with small intestinal adhesions and 20 intestinal volvulus. All horses received the same standard protocol of medication (flunixin-meglumine+xylazine).

Horses were premedicated with intravenous (IV) romifidine and anaesthesia was induced with guaifenesin+thiopental and was maintained with isoflurane as required. Lactated Ringer's solution (LRS) was given to all horses during anaesthesia. The outer parts of the resected intestine were harvested, divided in two portions: proximal and distal (a 20 cm segment that we assumed to be viable) to the stenosis, frozen in liquid nitrogen and stored frozen at -80°C.

Four adult horses destined for euthanasia for reasons unrelated to the cardio-vascular system or gastrointestinal tract were used as reference control.

All the studies were approved by the Complutense University Ethical Committee and adhered to the guidelines of Commission Directive 86/609/EEC (The Council Directive of the European Community) concerning the protection of animals used for experimental and other scientific purposes. The National legislation, in agreement with this Directive, is defined in Royal Decree n° 1202/2005.

### Isolation of tissue mitochondria

The tissue samples were placed in an ice-cold homogenization buffer (0.32 M sucrose, 1 mM K^+^EDTA, and 10 mM Tris-Cl, pH 7.4), containing 2.5 mg/ml of fatty acid-free bovine serum albumin (BSA) and 0.3 mM phenylmethylsulfonyl fluoride (PMSF, a protease inhibitor). The sections were minced and homogenized with 2 ml of homogenization buffer per gram of tissue, using a Teflon-glass homogenizer. The homogenate was centrifuged at 500 × g at 4°C for 5 min. The pellet was washed with the homogenization buffer and centrifuged at 500 × g for 5 min (4°C). The supernatant was centrifuged at 10,000 × g at 4°C for 15 min. The mitochondrial pellet was washed once and then resuspended in homogenization buffer. After centrifugation the supernatant was used for cytochrome *c *determination. The mitochondrial pellet was incubated with lyses buffer and used for NO and CO determination.

### Biochemical determinations

For GSH assessment, a specific colorimetric method was used [21]. Briefly, glutathione was sequentially oxidized by 5-5' dithio-bis (2-dinitrobenzoic acid) (DTNB) and reduced by NADPH in the presence of glutathione disulfide reductase, which results in the formation of 5-thio-2-nitrobenzoic acid (TNB). The rate of TNB formation is measured spectrophotometrically at 412 nm.

Cellular content of adenosine tri phosphate (ATP) and lipid hydroperoxides (LPO) were measured by spectrophotmetry using commercially available kits (Sigma, St. Louis, Missouri, USA; Camille Biochemical Company, Thousand Oaks, CA, USA, and).

For ATP determination, a portion of tissue was homogenised in tris-EDTA buffer, pH 7.75, containing 0.3% trichloroacetic acid, and centrifuged (3000 g, for 10 minutes). ATP measurement was based on two consecutive reactions; in the first one, ATP is transformed to adenosyl diphosphate (ADP) and 1,3-diphosphoglycerate in the presence of 3-phosphoglycerate phosphokinase); in the following reaction, catalyzed by glyceraldehyde-phosphate-dehydrogenase, 1,3-diphosphoglycerate in the presence of NADH+H is transformed in glyceraldehyde-3-P and NAD+P. The reduction of absorbance at 340 nm due to the oxidation of NADH to NAD is proportional to the amount of ATP in the sample [22,23].

Lipid hydroperoxides were extracted from the sample into chloroform before performing the assay. Briefly, tissue was homogenized in buffer phosphate saturated methanol and centrifuged, 3,000 × g, for 10 minutes. Five hundred microliters of the supernatant were aliquot into a glass test tube and an equal volume of cold chloroform was added and mixed thoroughly by vortexing, and centrifuged again at 1,500 × g for 5 minutes at 0°C. Bottom chloroform layer was collected and transferred to another test tube for LPO measurement. The basis of the LPO determination is the reaction of hydroperoxides with 10-N-methylcarbamoil-3,7-dimethylamino-10-10-fenotiacine, catalyzed by haemoglobin, which leads to methylene blue formation. The methylene blue formed was then measured colorimetrically [22,23].

NO release was measured by the Griess reaction as NO_2 _concentration after NO_3 _reduction to NO_2_. Briefly, samples were deproteinized by the addition of sulfosalicylic acid, were then incubated for 30 min at 4°C, and subsequently centrifuged for 20 minutes at 12,000 *g*. After incubation of the supernatants with *Escherichia coli *NO_3 _reductase (37°, 30 min), 1 ml of Griess reagent (0.5% naphthylenediamine dihydrochloride, 5% sulfonilamide, 25% H_3_PO_4_) was added. The reaction was performed at 22°C for 20 min, and the absorbance at 546 nm was measured, using NaNO_2 _solution as standard. The measured signal is linear from 1 to 150 μM (*r *= 0.994, *P *< 0.001, n = 5), and the detection threshold is ~2 μM.

To quantify the amount of CO released, the ratio of carboxy-haemoglobin after haemoglobin addition was measured. Haemoglobin (4 μM) was added to samples and the mixture was allowed to react for 1 min, to be sure of a maximum binding of CO to haemoglobin. Then, samples were diluted with a solution containing phosphate buffer (0.01 mol/L monobasic potassium phosphate/dibasic potassium phosphate, pH 6.85) containing sodium dithionite, and after 10 min at room temperature, absorbance was measured at 420 and 432 nm against a matched curve containing only buffer.

MAT activity was assayed as described by Cantoni [24,25]) and Duce [25]. Briefly, the incubation mixture consisted of 100 mM Tris-HCl (pH 7.8), 200 mM KCl, 10 mM MgCl_2_, 1 mM dithiothreitol,, 5 mM ATP and 5 mM L-methionine containing 0.2 μCi/ml methyl-L-[^3^H]methionine (Radiochemical Center, Amersham, Buckinghamshire, UK). The reaction was initiated by the addition of the sample and 30 min later was stopped with cold distilled water. The incubation mixture was immediately applied to a 2 ml Dowex AG 50 W column. The column was washed with 20 ml distilled water and the [^3^H]SAMe formed was then eluted with two fractions of 3 ml of 3 N ammonium hydroxide. The s-adenosyl-L-methionine formed was determined by counting in 10 ml F-1 Normascint scintillation liquid (Scharlau). The reaction was linear with time for at least 30 min.

Phospholipid Met Tase was determined as described elsewhere [25]. This method is based on the determination of the incorporation of [^3^H]methyl groups from S-adenosyl-L-(methyl-^3^H)methionine (Radiochemical Center, Amersham, Buckinghamshire, UK) into phospholipids. The reaction mixture contained 10 Mm 4,2-hydroxyethyl-1-piperazine ethanesulfonic acid (HEPES) (pH 7.3), 4 mM dithiothreitol, 5 mM MgCl_2_, 100 μM S-adenosyl methionine, 2 μCi S-adenosyl-L-(methyl-^3^H)methionine and 50 μl of sample. The reaction was initiated by the addition of a mixture of the labelled and unlabeled S-adenosyl methionine and terminated by pipetting 100 μl assay mixture into 2 ml chloroform/methanol/2 N HCl (6:3:1, v/v/v) for lipid extraction. The chloroform phase was washed with 1 ml 0.5 M KCl in 50% methanol. After washing, 0.6 ml of the chloroform phase was pipetted into a counting vial, dried at room temperature, dissolved into 5 ml Normascint-11 scintillation liquid (Scharlau) and counted.

Results are expressed in relation to the concentration of tissue proteins to correct differences in the amount of cell per surface, secondary to intestinal wall dilation. Protein determination was performed by the Bradford method. The basis of this method is the addition of Coomassie brilliant blue dye/colorant to proteins. This union induces a shift in maximum dye absorbance from 465 to 595 nm. Absorbance is measured at 595 nm, comparing to a known standard curve.

Reproducibility within the assays was evaluated in three independent experiments. Each assay was carried out with three replicates. In all assays, the intra-assay coefficient of variation was <5%, and the inter-assay coefficient of variation was <6%.

### Statistical analysis

Results are expressed as the mean ± SEM. Mean comparison was done by the Kuskal-Wallis test followed by a Mann Whitney test; a confidence level of 95% (p < 0.05) was considered significant.

## Results

**Figure 1:**
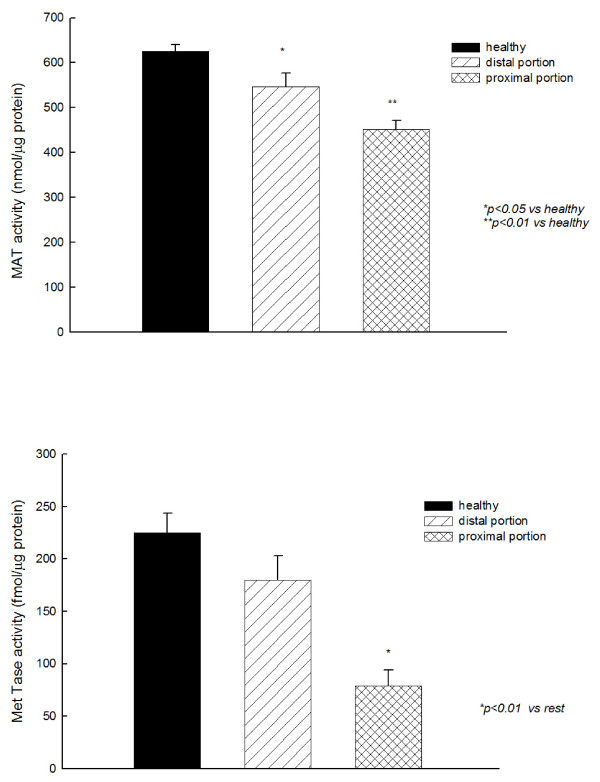
**Methionine adenosyl transferase (MAT) and methyl transferase (MetTase) activities in jejunum of colic horses**.

MAT activity was reduced in both, distal and proximal portions of jejunum obtained of colic horses, compared with the MAT activity observed in the intestine of healthy horses (Fig. 1A). Met Tase activity was also lower in the portion of intestine proximal to the stenosis compared to the distal group, however, no differences were observed between distal portion and healthy horses (Fig. 1B).

**Figure 2:**
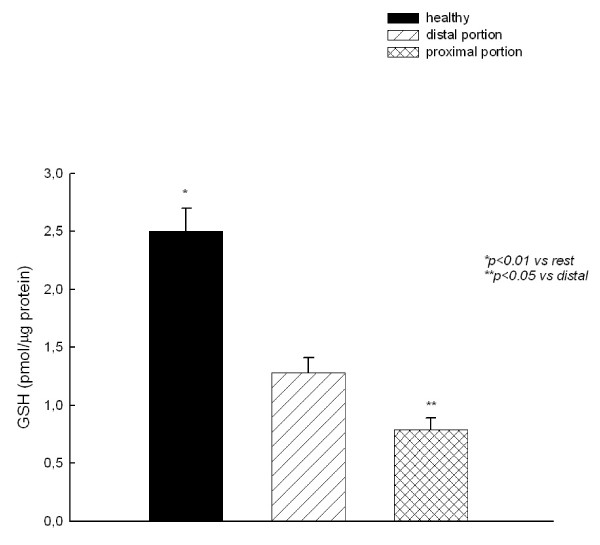
**Glutathione (GSH) content of jejunum homogenates**.

**Figure 3:**
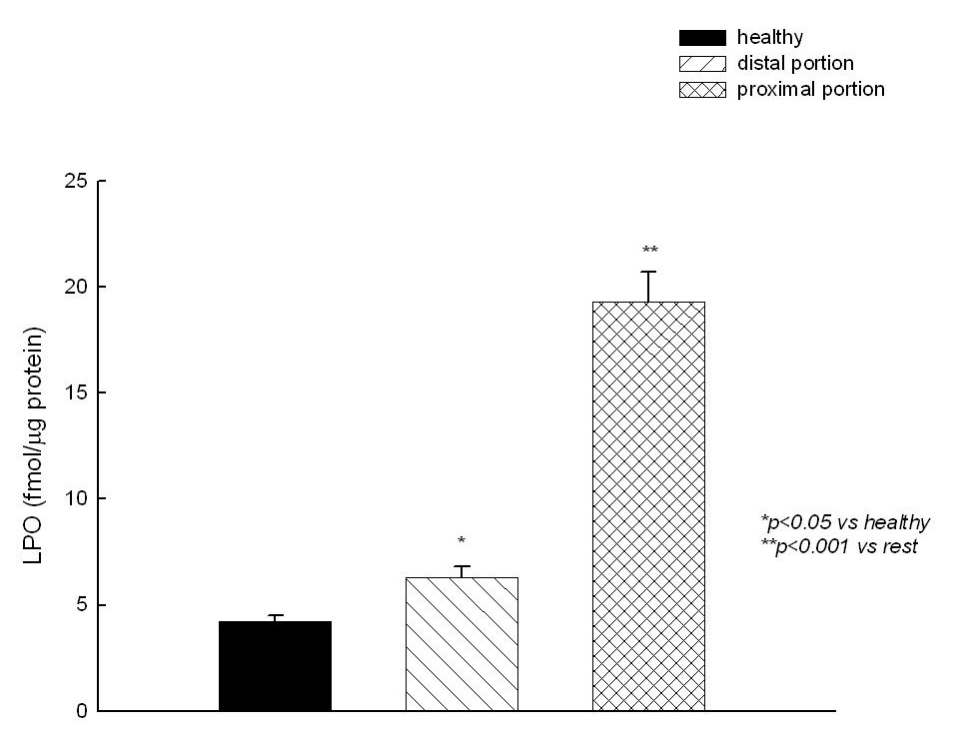
**Lipid peroxides (LPO) content of jejunum homogenates**.

GSH content of tissue distal to the stenosis was significantly lower than that of the healthy horses, and a further reduction was observed in the proximal portion (Fig. 2). By contrary, LPO levels were lower in intestinal tissue of healthy horses compared with colic horses. LPO levels were higher in cells from the proximal portion as compared to those from the distal portion (Fig. 3).

**Figure 4:**
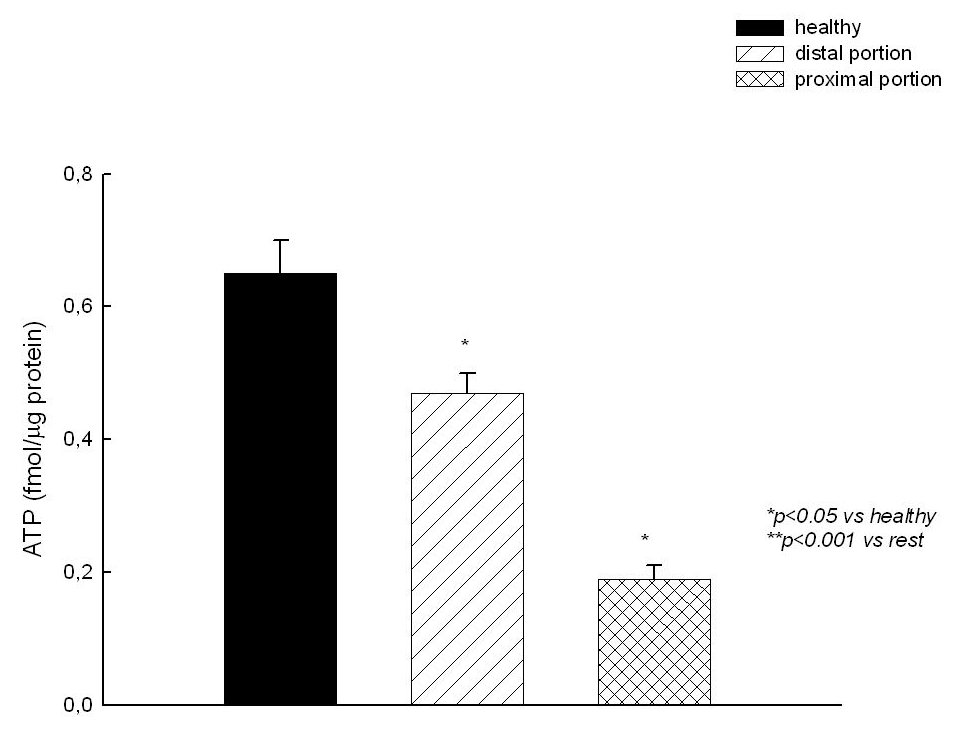
**Adenosyl triphosphate (ATP) content jejunum homogenates**.

As shown in figure 4, ATP content followed a pattern similar to that of GSH. ATP was reduced in the proximal portion to the stenosis compared to the distal one.

**Figure 5:**
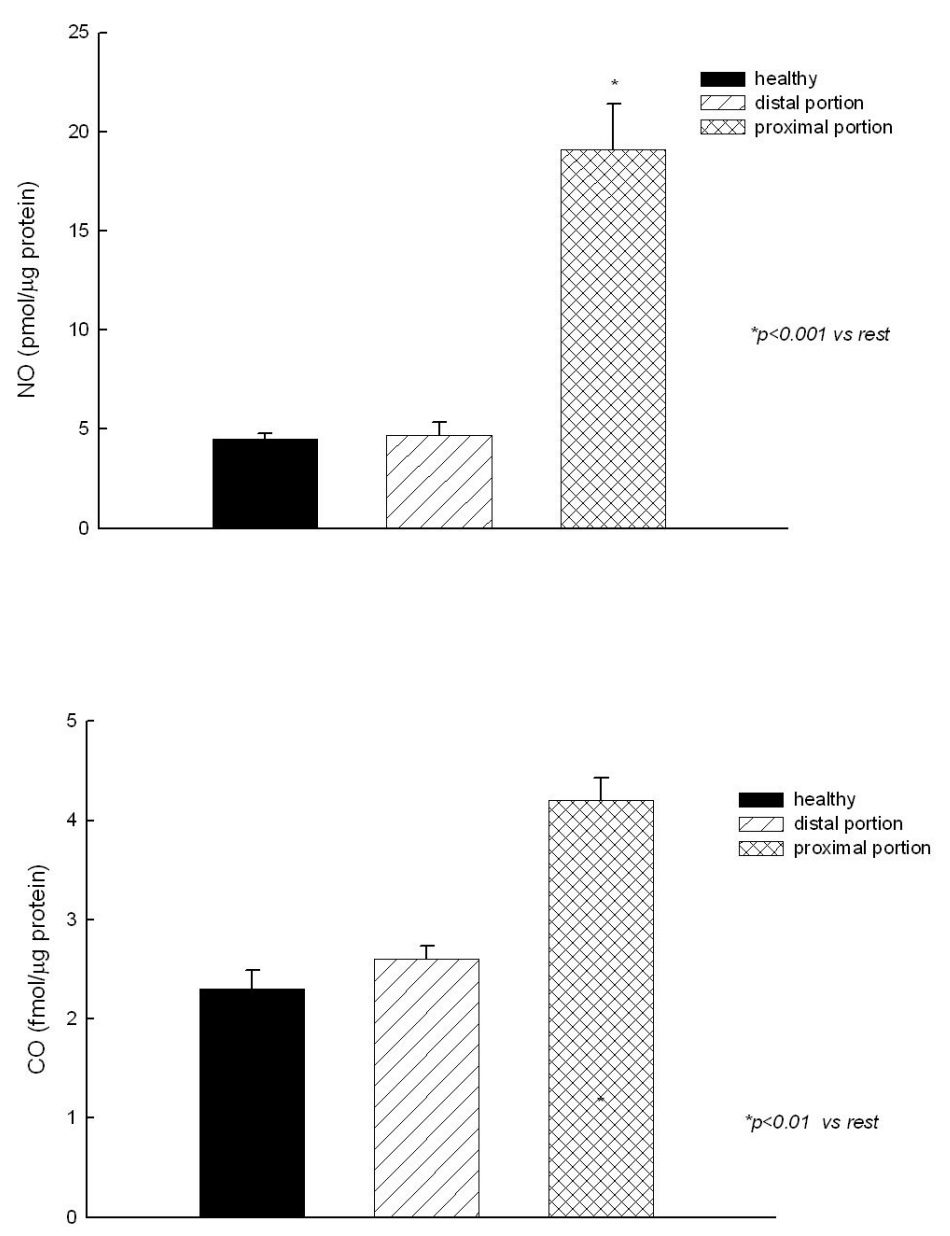
**Nitric oxide (NO) and carbon monoxide (CO) level in jejunum homogenates**.

The NO and CO content (released by) in mitochondria gave higher values in the proximal portion as compared with the distal group (Fig. 5). No differences were observed between distal portion and healthy horses.

## Discussion

It is generally accepted that the intestinal mucosa is extremely sensitive to ischemia. Ischemia injury causes misdistribution of blood flow, damage to endothelium, coagulation abnormalities, and aggregation of platelets and neutrophils. The activation of neutrophils leads to the release of reactive oxygen species (ROS) including superoxide anion (O_2_^-^) and H_2_O_2_.

Free radicals generated by polymorphonuclear leucocytes, macrophages and other cells, may have a role in intestinal dysfunction secondary to ischemia [1,5,26]. One of the most immediate actions of free radicals is the peroxidation of membrane lipids which is implicated as the underlying cause of cell injury in many conditions involving oxidative stress. Although the mechanisms of lipid peroxidation have been extensively studied, controversy still remains as to the critical and irreversible steps leading to cell injury and the therapeutic potential of intervention [27]. In this study, we measured LPO levels as damage indices in the intestine. We found a considerable increase in the intestinal tissue levels of LPO in the outer parts of resected intestine segments isolated from colic horses compared with those isolated from healthy horses, and this increase was more marked in the proximal portion to the stenosis compared with the distal portion, indicating that free radicals, secondary to intestinal ischemia, promote the peroxidation of intestinal lipids. Interestingly, higher LPO levels were also found in the distal (macroscopically viable) portion of intestine resected compared with those isolated from healthy horses. We can speculated that the ischemic intestine releases proinflammatory molecules, such as hydrogen peroxide (H_2_O_2_), superoxide radicals (O_2_^-^), cytokines (e.g., tumour necrosis factor-α), and arachidonic acid metabolites into the portal and systemic circulation, all of which can induce direct tissue damage. However, these molecules are also potent activators of leukocytes and thereby promote their sequestration to the neighborhood, increasing damage to intestine.

Several inflammatory mediators can be released during equine colic [3,28]. Nitric Oxide (NO) is one molecular mediator involved in both the inflammatory response [29] and oxidative damage [8,30]. During defence reactions there seems to be a close relationship between both free radicals and NO production. NO reacts with oxygen to yield peroxynitrites, which is a strong oxidant.

In accordance with previous reports, which show that iNOS activity and NO release are increased after ischemia injury in several tissues [31-33], in the present study NO release to the mitochondrial fraction was found to be increased in the portion of jejunum proximal to the stenosis compared with the distal group. It is likely that NO is produced in the intestinal tissue during different processes such as vascular regulation and host defence. In addition, it is conceivable that NO produced by any cell may exert paracrine effects in neighboring cells, then amplifying tissue damage.

GSH is probably the most important cellular antioxidant. There is evidence for an evolutionary link between GSH and eukaryotic cell metabolism [34] indicating that GSH evolved as a molecule that protects cells against oxygen toxicity. Intracellular GSH acts both as a nucleophilic "scavenger", converting electrophylic compounds to thioether conjugates, and as a substrate in the GSH peroxidase-mediated destruction of hydroperoxides [35]. GSH can prevent covalent binding of reactive metabolites to critical cellular macromolecules and lipid peroxidation [14], both of which are major mechanisms mediating cell injury and/or death. Glutathione (GSH) deficiency leads to severe degeneration of the epithelial cells of the jejunum and colon [16]. Administration of GSH have a protective effect on the gastrointestinal epithelium and may also serve as a good source of cysteine for intracellular GSH synthesis in the gastrointestinal tract and in other tissues [16]. In this study, a reduction in GSH levels in the portion of jejunum proximal to the stenosis was observed. This fact could be either cause or consequence of the increase in the intestinal ischemia-related oxidative/antioxidative imbalance.

SAMe, an endogenous cellular metabolite that acts as methyl donor in most of biological transmethylation reactions, is also able to act as an antioxidant and free radical scavenger in vitro [36]. It has also shown to exert protective effects on different experimental pathological models, in which free radicals and ROS are involved, such as brain ischemia-reperfusion [37], cytokine-induced toxicity [38], liver cirrhosis [39-42], cholestatic liver disease [43-46] and in the exposure to hepatotoxic agents [17]. Thus it seemed interesting to look into the implication of this molecule during equine colic, during which, release of free radicals seems to be involved [1,5].

A decrease in MAT activity in the outer parts of resected jejunum segments isolated from colic horses compared with those isolated from healthy horses has been found in this study, and this decrease was more marked in the proximal portion of stenosis compared with the distal portion. This finding is in accordance with previous studies in humans and experimental animals, in which a decrease in liver-specific MAT activity in several pathological situations was observed [17,25,39]. However, to our knowledge this is the first work showing a decrease in MAT activity in the intestine of colic horses with intestinal ischemia. This reduction in MAT activity would lead to a decrease in SAMe synthesis, which would affect many essential metabolic pathways in which SAMe is involved, such as GSH synthesis. Oxidative stress could be involved in this intestinal ischemia-related decrease in MAT activity, since it is known that both ROS [47,48] and NO [49] are able to inactivate liver MAT. This situation would lead to a self-perpetuating cycle, in which the free radicals generated would induce a GSH depletion, thus increasing oxidative stress that would inactivate MAT, which would further reduce SAMe and GSH synthesis [50,51].

On the other hand, in our study intestinal ischemia induced an increase in cellular oxidative stress (as shown by the increase in LPO content) and NO release, and these factors could account for the reduction in MAT activity. This decrease in MAT activity has been proposed to be an adaptative mechanism to spare ATP, whose levels are usually compromised under pathological situations, by reducing its consumption in SAMe and GSH synthesis and leaving it available for other basic cellular functions [17]. In this study, a reduction in GSH levels in the portion of jejunum proximal to the stenosis was also found, which could be both cause and consequence of the decrease in MAT activity.

SAMe can also contribute to preserve cell membrane integrity, preventing membrane lipid peroxidation and maintaining phospholipid methylation, and membrane fluidity. In this study, we also found a colic-associated decrease in MetTase activity, a fact that has also been found in other pathological situations [25,52]. Given that Met Tase plays an important role catalyzing the methylation of phosphatidyl ethanolamine to produce phosphatidyl choline (PC), which is an essential molecule for cell membrane integrity, we can hypothesized that this decrease in Met Tase could contribute to ischemia-induced intestinal tissue injury.

It is known that impairment in mitochondrial function, may lead to an increase in the rate of ROS production in mitochondria and this fact could be a major mechanism for the intestinal ischemia-related increase in oxidative damage in colic horses. Impairment in structure and/or mitochondrial function may lead to a reduction of the energy supply to the cells. These observations are in accordance with our present results, in which a decrease in ATP content of the intestinal tissue resected from colic horses, compared with those obtained from healthy horses. Accumulation of oxidative damage could be involved in this phenomenon, since oxidative stress is able to inhibit mitochondrial respiration [6] leading to organ dysfunction and cell death.

CO is a physiologically synthesized molecule that shares some of the mechanisms of action and physiological effects of NO [9,53]. The main endogenous source of CO is heme metabolism by heme-oxygenase (HO) [9,53]. This HO-CO pathway has been recently proposed to be involved in the defence against oxidative stress and the deleterious effects of NO, since it removes the cytotoxic free heme, and produces some molecules with antioxidant and anti-inflammatory effects, such as biliverdin and CO [9,54-56]. Therefore, this pathway could be activated to counteract an excess of oxidant and inflammatory agents [9]. The present study shows that intestinal ischemia induces an increase in local CO production in intestine, and this fact could mean that this defence mechanism has been activated by the increase in colic-associated ROS and proinflammatory molecules, such as NO.

## Conclusion

In conclusion, our results suggest that intestinal ischemia in horses can be accompagnied with an oxidative/antioxidative imbalance. This effect could be mediated, at least in part by impairment in glutathione and/or SAMe metabolism, suggesting a possible application for these molecules in the preventive and/or therapeutic approach to intestinal ischemia-induced damage. Further investigation is needed to elucidate their efficacy and establish if they can be used clinically.

## Abbreviations

ADP: adenosyl diphosphate; ATP: adenosine triphosphate; BSA: bovine serum albumin; cGMP: cyclic-guanosyl monophosphate; CO: carbon monoxide; DTNB: 5-5' dithio-bis (2-dinitrobenzoic acid); EDTA: ethylene diamine tetra acetic; GSH: glutathione; HEPES: 4,2-hydroxyethyl-1-piperazine ethanesulfonic acid; HO-1: heme-oxygenase 1; LPO: lipid hydroperoxides; MAT: methionine-adenosyl-transferase; MetTase: methyl-transferases; NAD: oxidized form of nicotin adenin dinucleotide; NADH+H: reduced form of nicotin adenine dinucleotide; NADPH+H: reduced form of nicotin adenine dinucleotide phosphate; NO: nitric oxide; P: phosphate group; PMSF: phenylmethylsulfonyl fluoride; RNS: reactive nitrogen species; ROS: reactive oxygen species; SAMe: S-Adenosyl methionine; sGC: soluble-guanilyl cyclase; TNB: 5-thio-2-nitrobenzoic acid.

## Authors' contributions

All authors have participated sufficiently in the work to take public responsibility for its content. GM carried out the surgery, took the samples and participated in NO and CO determination. WM participated in the surgery and performed the statistical analysis. PC participated in the surgery and drafted the manuscript. CG carried out MAT, GSH and Met Tase determination. EV conceived the study, and participated in its design and coordination and helped to draft the manuscript. All authors read and approved the final manuscript.
